# Harnessing machine learning in contemporary tobacco research

**DOI:** 10.1016/j.toxrep.2024.101877

**Published:** 2024-12-19

**Authors:** Krishnendu Sinha, Nabanita Ghosh, Parames C. Sil

**Affiliations:** a Jhargram Raj College, Jhargram 721507, India; b Maulana Azad College, Kolkata 700013, India; c Division of Molecular Medicine, Bose Institute, Kolkata 700054, India

**Keywords:** Machine learning, Algorithms, Smoking, Tobacco, Vapes, Cancer

## Abstract

Machine learning (ML) has the potential to transform tobacco research and address the urgent public health crisis posed by tobacco use. Despite the well-documented health risks, cessation rates remain low. ML techniques offer innovative solutions by analyzing vast datasets to uncover patterns in smoking behavior, genetic predispositions, and effective cessation strategies. ML can predict smoking-induced non-communicable diseases (SiNCDs) like lung cancer and postmenopausal osteoporosis by identifying biomarkers and genetic profiles, generating personalized predictions, and guiding interventions. It also improves prediction of infant tobacco smoke exposure, distinguishes secondhand and thirdhand smoke, and enhances protection strategies for children. Data-driven, personalized approaches using ML track real-time data for personalized feedback and offer timely interventions, continuously improving cessation strategies. Overall, ML provides sophisticated predictive models, enhances understanding of complex biological mechanisms, and enables personalized interventions, demonstrating significant potential in the fight against the tobacco epidemic.

## Highlights

• Machine learning (ML) transforms tobacco research by analyzing vast datasets for innovative insights.
• ML predicts smoking-induced diseases via biomarkers and genetic profiling.
• ML improves secondhand and thirdhand smoke predictions, enhancing child protection.
• Data-driven ML offers personalized interventions for effective cessation strategies.
• ML advances understanding of complex biological mechanisms in smoking-related diseases.

## Graphical Abstract

**Figure:**
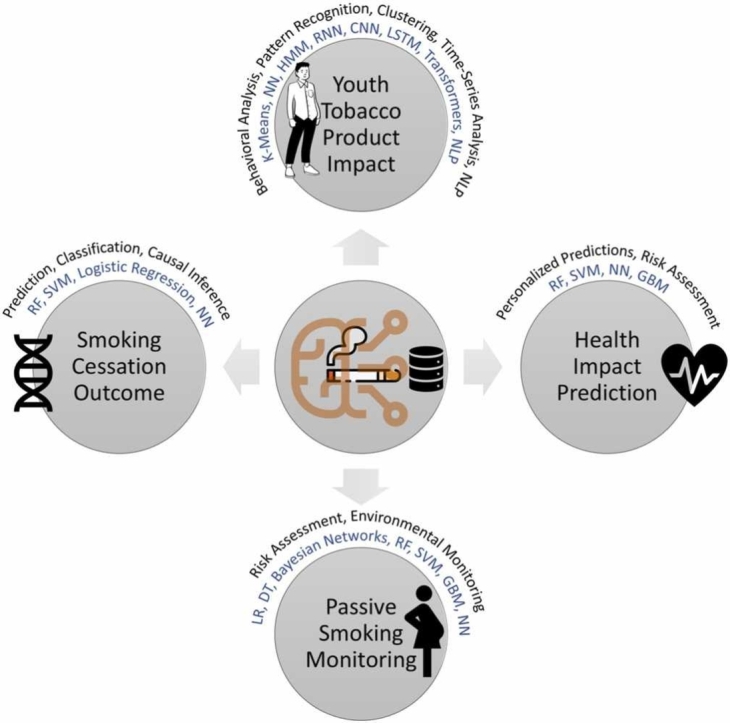

## 1 Introduction

Tobacco smoking poses a significant public health threat due to its immediate and long-term detrimental effects on physical health, including increased risk of respiratory infections, various cancers, cardiovascular diseases, and weakened immune function [1], [2], [3], [4]. In 2020, the World Health Organization (WHO) reported that 36.7 % of men and 7.8 % of women, totalling 22.3 % of the global population, used tobacco [5]. Tobacco use is the leading preventable cause of death worldwide, causing 8 million deaths annually, including 1.2 million non-smokers affected by passive smoking [2], [6]. Without effective tobacco control efforts, this number is projected to increase to 18.3 million by 2030 [7]. In the U.S., approximately 14 % of adults, or 34 million people, are active combustible tobacco users, who could gain up to 10 years of life by quitting [8]. Prevention is critical in addressing the tobacco use pandemic [9]. Around 70 % of people who use combustible tobacco want to quit but typically need an average of 6 attempts to achieve lasting abstinence, with nicotine replacement therapy (NRT) and counselling being effective [8]. Tobacco use is a complex behavior influenced by genetic factors (up to 50 %) and various socio-environmental factors like peer influence, workplace culture, social gatherings, media and advertising, stressful situations, community regulations, presence of triggers etc [8]. Additionally, electronic nicotine delivery systems (ENDS), such as e-cigarettes, are becoming a public health concern, particularly among adolescents. Although ENDS were initially developed to help people quit tobacco, their rapid and unregulated growth has posed significant risks to public health, leading to increased scrutiny and regulation, especially regarding advertising and sales on social media [10].

In recent years, the shift from traditional computational methods to machine learning (ML) models has been driven by the availability of vast amounts of data [11], [12]. ML is now making significant impacts in fields such as psychology, medicine, and public health [12]. Researchers are using ML to tackle complex issues in tobacco research, such as understanding psycho-genetic predispositions to tobacco addiction, analyzing intricate behavioral patterns in people who use tobacco, providing personalized digital tools to assist in quitting, and monitoring unregulated vaping trades on social media [12], [13]. These advancements, once considered unimaginable, are now becoming integral in the fight against the tobacco epidemic, highlighting the transformative potential of next-generation ML algorithms and big data research [14].

**Fig. 1:**
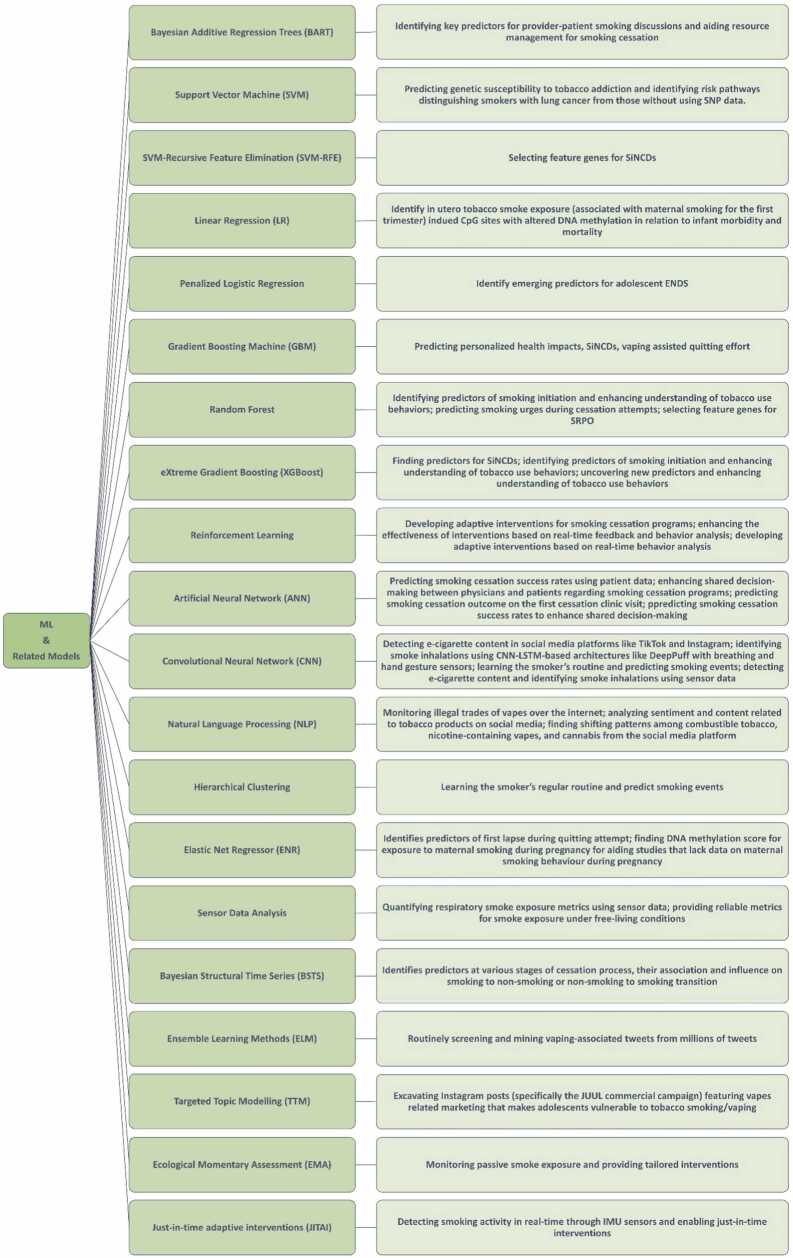
A hierarchical chart illustrating how different ML approaches have been used to solve various tobacco research problems.

In this review, we aim to formulate four comprehensive questions (Table 1) shaping contemporary tobacco research and identify the limitations of traditional methodologies. By exploring the transformative potential of ML, we demonstrate how it can effectively address these challenges and fill existing research gaps. Our goal is to provide researchers with a comprehensive, up-to-date overview of ML applications in tobacco research, empowering them to leverage these insights in their work (Fig. 1; Table 2). We believe this review will serve as a valuable resource, fostering innovation and collaboration within the research community.

**Table 1:** Key research questions in tobacco control, associated problem types, applicable machine learning models, and potential outcomes.

Research Question	Problem Type	ML Model Type	Model Description	Examples of Previous Work	Potential Outcomes
How can we predict the individual health impacts of smoking?	Personalized Predictions, Risk Assessment, Classification, Predictive Analytics	Random Forest (RF), Support Vector Machine (SVM), Neural Networks (NN), Gradient Boosting Machines (GBM)	RF: Ensemble of decision trees for classification, SVM: Hyperplane classification, NN: Complex pattern recognition, GBM: Sequential model building	[14], [15], [16], [17], [18], [19], [20], [21], [22], [23], [24], [25], [26], [27], [28], [29], [30], [31], [32], [33]	Accurate individual health risk assessments, personalized intervention strategies, improved health outcomes
How can we accurately assess and monitor passive smoke exposure?	Risk Assessment, Environmental Monitoring, Predictive Analytics, Anomaly Detection	Logistic Regression, Decision Trees, Bayesian Networks, Gradient Boosting Machines (GBM), Random Forest, Neural Networks, Support Vector Machine (SVM)	Logistic Regression: Probability estimation, Decision Trees: Simple classification, Bayesian Networks: Probabilistic graphical models, GBM: Sequential model building, RF: Ensemble of trees, NN: Complex pattern recognition, SVM: Hyperplane classification	[34], [35], [36], [37], [38], [39]	Proactive protection measures, real-time exposure monitoring, tailored interventions, improved monitoring of exposure levels, better public health strategies
How to predict and improve smoking cessation outcome?	Prediction, Classification, Causal Inference	Random Forest (RF), Support Vector Machine (SVM), Logistic Regression, Neural Networks (NN)	RF: Ensemble of decision trees, SVM: Hyperplane classification, LR: Probability prediction, NN: Complex patterns recognition	[40], [41], [42], [43], [44], [45], [46], [47], [48], [49], [50], [51], [52], [53], [54], [55], [56], [57], [58], [59], [60]	Improved prediction models for intervention effectiveness, targeted cessation programs, higher cessation rates, tailored support programs, reduced relapse rates
What is the impact of evolving tobacco product landscape on youth and how to mould intervention strategies accordingly?	Behavioral Analysis, Pattern Recognition, Clustering, Time-Series Analysis, Social Media Analysis, Natural Language Processing, Impact Assessment, Intervention Design	K-Means Clustering, Neural Networks (NN), Hidden Markov Models (HMM), Recurrent Neural Networks (RNN), Convolutional Neural Networks (CNN), Long Short-Term Memory (LSTM), Transformers, Natural Language Processing (NLP), Clustering Algorithms	K-Means: Data partitioning, NN: Complex pattern recognition, HMM: State transitions, RNN: Sequence modeling, CNN: Image data processing, LSTM: Sequence data processing, Transformers: Advanced NLP models, NLP: Understand, interpret, and manipulate human language, Clustering: Identifying youth segments based on behavior	[61], [62], [63], [64], [65], [66], [67], [68], [69], [70], [71], [72], [73], [74], [75], [76], [77], [78], [79], [80], [81], [82], [83]	Insights into youth behavioral patterns, development of effective youth-focused interventions, better regulation and tracking of vaping products, reduced initiation rates

**Table 2:** Comprehensive glossary.

Term	Definition
Adaptive Boosting (AdaBoost)	A machine learning meta-algorithm that combines weak learners to create a strong learner, used for classification and regression tasks.
Artificial Neural Networks (ANN)	A machine learning model inspired by the structure and function of biological neural networks, used for various tasks such as classification, regression, and prediction.
Bayesian Additive Regression Trees (BART)	A non-parametric Bayesian approach to modeling data, used in tobacco research for recognizing key determinants promoting smoking cessation discussions.
Convolutional Neural Networks (CNN)	A type of artificial neural network used in image recognition and processing, designed to automatically and adaptively learn spatial hierarchies of features.
Decision Tree (DT)	A supervised machine learning algorithm used for classification and regression tasks.
Deep Learning	A subset of machine learning involving neural networks with many layers (deep neural networks) that learn from large amounts of data. Includes models like CNNs for visual analysis and LSTMs for sequential data processing.
DeepPuff	A CNN-LSTM-based deep learning architecture quantifying Respiratory Smoke Exposure Metrics (RSEM) by detecting smoke inhalations via sensors.
DNA Methylation	A process by which methyl groups are added to DNA molecules, affecting gene expression, used in ML models to identify fetal exposure to maternal smoking.
Ecological Momentary Assessment (EMA)	A research method that involves collecting real-time data on participants' behaviors, symptoms, and contexts in their natural environments.
Electronic Nicotine Delivery System (ENDS)	Also known as e-cigarettes, they are becoming a public health concern, particularly among adolescents, despite being initially developed to help people quit tobacco.
eXtreme Gradient Boosting (XGBoost)	A scalable and efficient implementation of gradient boosting, a machine learning technique that produces a prediction model in the form of an ensemble of weak prediction models.
Gradient Boosting Machine (GBM)	A machine learning technique that produces a prediction model in the form of an ensemble of weak prediction models, typically decision trees.
Hierarchical Cluster Analysis (HCA)	An unsupervised machine learning algorithm used for clustering data.
Just-in-time adaptive interventions (JITAI)	Interventions that aim to provide the right type/amount of support at the right time by adapting to an individual's changing internal and contextual state.
k-Nearest Neighbors (KNN)	A non-parametric method used for classification and regression, where the output is a class membership or a property value for an object based on the k nearest training examples in the feature space.
Least Absolute Shrinkage and Selection Operator (LASSO)	A regression analysis method that performs both variable selection and regularization in order to enhance the prediction accuracy and interpretability of the resulting statistical model.
Light Gradient Boosting Machine (LGBM)	A highly efficient gradient boosting framework using tree-based learning algorithms, applied to pinpoint predictors of SHS-induced depression.
Linear Regression (LR)	A statistical method used to model the relationship between a dependent variable and one or more independent variables using a straight line.
Machine Learning (ML)	A shift from traditional computational methods driven by the availability of vast amounts of data, making significant impacts in fields such as psychology, medicine, and public health to tackle complex issues in tobacco research.
Nicotine Replacement Therapy (NRT)	Along with provider-patient discussion, is recommended for treating nicotine dependence, but the rate of success is not encouraging.
Principal Component Analysis (PCA)	A statistical procedure that uses an orthogonal transformation to convert a set of observations of possibly correlated variables into a set of values of linearly uncorrelated variables called principal components.
Random Forest (RF)	An ensemble learning method for classification, regression and other tasks, operating by constructing a multitude of decision trees at training time and outputting the class that is the mode of the classes or mean prediction of the individual trees.
Secondhand Smoke (SHS)	Smoke that non-smokers involuntarily inhale from nearby smokers, posing significant public health risks linked to respiratory infections, cardiovascular diseases, and cancer.
SHapley Additive explanation (SHAP)	A method for explaining ML model outputs by attributing each feature's contribution to predictions, used in assessing smoking cessation predictors.
Single Nucleotide Polymorphism (SNP)	A variation in a single nucleotide at a specific genome position, used in ML models to predict smoking behavior.
Smoking Induced Non-Communicable Diseases (SiNCDs)	Include stroke, heart disease, chronic respiratory diseases, cancers, etc., which can have significantly reduced death rates through early diagnosis, effective treatment, and smoking cessation.
Support Vector Machine (SVM)	A supervised machine learning model that analyzes data for classification and regression analysis.
Support Vector Machine Recursive Feature Elimination (SVM-RFE)	A feature selection algorithm that uses a SVM as the classifier and recursively removes features based on their weights until the desired number of features is reached.
t-Distributed Stochastic Neighbor Embedding (t-SNE)	A dimensionality reduction technique used for visualizing high-dimensional data.
Thirdhand Smoke (THS)	Residual contaminants that linger on surfaces and dust, posing health risks, especially to children.
Tobacco smoking	A highly complex behavior controlled by genetic predisposition and environmental parameters, posing serious health risks and being a leading preventable cause of death worldwide.
Weighted Gene Co-expression Network Analysis (WGCNA)	A systems biology method used to describe the correlation patterns among genes across microarray samples, with the goal of finding clusters (modules) of highly correlated genes, and relating these modules to external sample traits.

## 2 Literature search strategy

The literature search for this review employed a methodical approach to comprehensively cover advancements in ML-assisted tobacco research. Searches were conducted across databases including PubMed, Scopus, and Web of Science, using keywords like “machine learning”, “ML”, “tobacco research”, “smoking”, “tobacco”, “vaping”, “vape” and related terms. Boolean operators (AND, OR) were utilized to refine search queries and ensure inclusivity of relevant literature from January 2010 to June 2024. Additionally, hand-searching of reference lists and inclusion of grey literature such as conference proceedings supplemented the search. Criteria for inclusion encompassed studies employing ML techniques for analyzing tobacco-related data, including predictive modeling, pattern recognition, and sentiment analysis, focusing on tobacco use, cessation interventions, health outcomes, and policy evaluations. This methodical approach aimed to provide a comprehensive overview aligned with current research trends in ML applications within tobacco research.

## 3 A Brief account on ML

ML is the science of programming computer so that the computer can learn from data [15]. Conventional programming works by setting a directory of certain rules, which a computer follows to provide desirable outcome. In other words, the information goes in, rules applied, and the results come up. This approach turns out to be ridiculously challenging in the case of intricate dynamic situations like, assessing human emotions to addiction, extracting predictor for adolescent nicotine addiction, etc. On contrary, ML does not require setting of explicit rules to produce result. Through ML algorithms, computers extract pattern from input data and set instance specific rules. Historically ML is defined as the field of study that empowers computers to learn without being explicitly programmed [16]. In general, most of the complex ML challenges can be reduced to one of the four core problem types namely, Classification, Regression, Clustering and Rule extraction [17], [18]. Classification deals with labelling of discreate data points, meaning it assigned a class (e.g., smoker/non-smoker or ever-smoker/never-smoker) to a data point [19]. Based on these labelled datasets, a classification model gets train to allocate labels to new unlabeled data points. This can be understood as a discrimination problem, demonstrating the disparities or similarities among groups [18]. Regression deals with continuous numerical data points (generally floats). A regression model after being trained, can predict numerical outcome for new unpredicted data, like the average age of start smoking in a population under study [19], [20]. In clustering, unlabeled data can be split into groups based on similarity and additional measures of the inherent natural data structure. Identification of e-cigarettes related tweets from other non-specific tweets can be considered as clustering problem. Lastly, in the case of rule extraction problem, data is utilized for the extraction of propositional laws. Such rules are not usually directed, that mean, these methods acquire statistically acceptable associations among properties in the data, not essentially involving something that is being predicted [18]. An instance is the finding of the connection among the addiction of e-cigarettes, marijuana, or alcohol [19]. Commonly a vast majority of ML systems broadly fall under any of these four categories namely, supervised, unsupervised, semi-supervised, and reinforcement learning based on the degree of human supervision involved in model training to solve the four core problem types [15], [21], [22].

Supervised learning works with a group of labeled examples, i.e., training dataset. That can be mathematically represented as ${\left\{\left(x_{i},y_{i}\right)\right\}}_{i=1}^{n}$, where each element $x_{i}$ represents a feature vector, and the dimensionality of the dataset corresponds to the length of this vector [21]. Each dimensional value is a feature, represented as $x_{i}^{\left(j\right)}$ [32]. Feature defines an instance. All the feature vectors are typically loaded into a matrix layout where, individual row signifying a vector for one instance and individual column signifying all the instances’ values for that feature. As an instance, $x_{i}$ in one collection characterizes an adolescent vape user, then the first feature $x_{i}^{\left(1\right)}$could involve his age, the second feature $x_{i}^{\left(2\right)}$, could involve his education and so on. The label $y_{i}$ is generally an element to a finite set of classes or a real number indicating the label for the corresponding vector, e.g., ever-smoker or never-smoker [19], [21]. A class can be conceptualized as a category to which a feature vector belongs. In supervised learning the task of predicting a class is known as classification whereas, predicting a float is called regression [15], [21]. Decision Trees (DTs) and Random Forests (RFs), k-Nearest Neighbors (KNN), Support Vector Machines (SVMs), Linear Regression (LR), artificial neural networks (ANN) are some contextually relevant supervised learning algorithms. However, cumulatively it can be stated that the intent of a supervised learning algorithm is to utilize a labeled dataset to generate a model which in succession takes an unlabeled feature vector $x_{i}$ as input and outputs a label for that [21].

Unsupervised learning works with a set of unlabeled examples called training dataset. Mathematically speaking, the training dataset is represented as ${\left\{x_{i}\right\}}_{i=1}^{n}$, where every element $x_{i}$ is a feature vector. On contrary to supervised learning, unsupervised learning algorithm produce a model that accepts an unlabeled feature vector $x_{i}$ as input and either transforms it into another vector or into a scaler that can be utilized to resolve a practical problem [15], [21]. Clustering is a critical unsupervised learning technique appropriate for locating groups of analogous objects in a large pool of objects. A clustering model sends the ID of the cluster for every feature vector in the dataset. In other words, it puts label/ID to unlabeled data point based on its features[21]. Hierarchical Cluster Analysis (HCA), K-Means, Isolation Forest, t-Distributed Stochastic Neighbor Embedding (t-SNE), Principal Component Analysis (PCA) are few vital unsupervised learning clustering algorithms[15]. Examples of unsupervised learning in tobacco research contain discovering the topics of tobacco-related conversations on social media or finding possible subtypes of nicotine addiction by evaluating the brain MRI data of patients[23].

In the following sub-sections, a few widely adopted ML algorithms in the field of tobacco research are briefly discussed.

### 3.1 Naïve Bayes

Naïve Bayes is a probabilistic ML algorithms built on Bayes Theorem and largely used as a simple and powerful classifier, which assigns the maximum possible class of every single data point, matching to the description provided by the feature vector and assuming the independence of the predictors from each other [24], [25], [26], [27]. However, Naïve Bayes obtain remarkable results even under the situation of strong dependencies between predictors and works beautifully with small noisy datasets [25], [28].

### 3.2 Logistic regression

Logistic regressor (LR) can act as a binary classifier, which evaluates a data point’s probability of fitting into a particular class where a probability greater than 0.5 is considered as positive belonging to a particular class under observation [15]. Logistic regression model subtracts a weighted sum of the input features in addition to a bias term and, outputs the results [15]. A logistic sigmoid function outputs any float between 0 and 1. It is a very popular algorithm in ML studies.

### 3.3 k-Nearest Neighbours

k-Nearest Neighbours (KNN) is a supervised, non-parametric ML algorithm that identifies the closest neighbors to a given query point to determine its label or classification [29], [30], [31]. It is mostly used as a classifier, though it can perform a regression job. KNN presumes that, similar data points reside in vicinity. Distances between the query point and rest data points is calculated to infer the closest data points, and these distance metrics assist to form decision boundaries. These boundaries are commonly envisioned by Voronoi tessellation. Decision boundaries categorise query points in distinct classes. The k value is a critical hyperparameter that defines the number of neighbours to be checked for classifying a query point. As KNN algorithm commonly depend on Euclidian distance for the sake of classification, normalization generally boosts its performance drastically and the algorithm becomes sensitive to local data structure [29], [30], [32], [33]. Since KNN is part of a category of algorithms known for their lazy learning approach, its uses in an instance of substantially large dataset becomes prohibitively slow [29].

### 3.4 Decision trees

Decision Trees (DTs) are flexible non-parametric supervised learning algorithms applied intensively for classification and regression task [15]. Decision tree predicts the value of a target variable by inferring straightforward decision rules from predictors. A decision tree is a hierarchical construct starting at root node representing entire dataset, ending at terminal nodes representing classes resulting from tree analysis, and internal nodes in between, representing decision arguments [25]. Though individual decision trees have little, or no practical use and ensemble of rather uncorrelated trees form a random forest (RF) which is one of the most powerful and widely used ML model. This massive success of the ensemble method stands on a relatively basic perception of the ‘wisdom of crowd’. It is well-recognized that the combined responses from thousands of random individuals to a complex question often perform remarkably well, even rivaling the accuracy of an expert's answer [15]. Similarly, collective predictions of a collection of decision trees will often get improved forecasts in contrast to the best isolated predictor [15]. This collection of predictors is termed as ensemble and the process is entitled as the ensemble learning [15], [21], [25]. Random forest is a legendary ensemble method where, predictors are decision trees. To generate a prediction, the random forest forecasts the class that gets the most votes from individual decision trees [15]. Decision trees are exceptionally sensitive to the dataset for training and minor alteration in the dataset produce significant variation on tree morphology which satisfy the condition of independence between constituting tress of random forest. This is known as bootstrap aggregation or bagging. Bagging, together with few other methods like feature randomness, pasting etc., produce sufficiently random uncorrelated trees in a forest [15], [25], [33], [34].

### 3.5 Boosting algorithms

Boosting is an ensemble technique that combines multiple weak models, such as linear regression or shallow decision trees, to achieve high-quality predictions by training each model sequentially [35], [36]. In this process, each subsequent model addresses the shortcomings of the previous one. Adaptive Boosting (AdaBoost) and Gradient Boosting are two widely recognized boosting techniques, both of which iteratively add weak predictors to the ensemble, with each predictor improving on its predecessor [35]. eXtreme gradient boosting (XGBoost), a popular implementation of gradient-boosted decision trees, is an optimized, distributed gradient boosting library known for its flexibility, efficiency, and portability. It has been extensively utilized in tobacco research [37], [38].

### 3.6 Support vector machines

Support Vector Machine (SVM) is a prevailing, extensively used ML algorithm with the capability of handling high dimensional, non-linear, complex, noisy data and can be used both as classifier and regressor [25]. SVM works by deploying a hyperplane with N-1 dimension in an N-dimensional space (where N is the number of features) that explicitly classifies the data points based on their position in respect to the hyperplane. For a two-dimensional data, a hyperplane can be visualized a one-dimensional line that acts a decision boundary. The data points on either side of the boundary can be assigned distinct classes [15], [25]. Multiple hyperplanes may exist to separate two classes of data points, but the hyperplane with the maximum margin is considered ideal. Essentially, in the simplest scenario, an SVM classifier can be visualized as creating the widest possible "street" that separates two classes in a two-dimensional plane [15], [39]. Adding new data points outside this street does not affect the decision boundary, as it is solely determined by the data points situated at the street's edges [15], [40]. These critical data points, referred to as support vectors, define the hyperplane's orientation and position. For complex datasets, such as those used in vape-related sentiment analysis or smoking pattern studies, linear separation is not feasible [23], [41], [42]. In such cases, SVM employs kernel techniques to handle the complexity. Kernels enable the resolution of non-linear problems by transforming non-linearly separable data into a higher-dimensional space where linear separation becomes possible. Kernel functions achieve this transformation, making the data linearly distinguishable in the new space. Kernel-based methods, including SVM, are highly effective in high-dimensional classification tasks due to their capacity to generalize within such spaces [25], [39], [40], [43].

### 3.7 Artificial neural networks

Artificial neural network (ANN) is a deep learning algorithm made up of artificial neurons or linear units which simulate the basic architecture of brain in living organism. Linear units are nothing but linear equations which takes input to process with assigned weight along with prefixed bias and gives the output [44]. Neural networks typically unify their linear units into layers and linear units taking a common set of inputs establish a dense layer which by stacking together forms a deep neural network [45]. Despite simple transformation performed by each layer, a deep neural network tries to approximate complex thinking process of brain [45]. Nevertheless, dense layer composed of linear units are not suitable for real world problem as it can never go outside the linearity and thus an activation function like rectified linear unit (ReLU) is added in between two dense layers to import non-linearity into the system and makes stacking effective [44], [45], [46], [47].

Inspired by the concept of an artificial neuron, multiple neurons can be connected to form a network, where the output of one neuron serves as the input for another. The input layer receives data from external sources, typically in a vectorized or tensor format, while the output layer provides the final results. In regression tasks, the output layer functions as a simple linear unit, whereas for classification tasks, it incorporates an activation function [25], [45]. The intermediate nodes, situated between the input and output layers, are referred to as hidden layers because their outputs are not directly accessible to the user [15], [44]. Deep learning represents a specialized form of neural networks, usually consisting of a large number of densely interconnected layers [48]. Its core principle lies in hierarchical information processing, where each layer of neurons works to derive increasingly meaningful representations of the data. Lower layers capture basic features, while subsequent layers combine these simpler elements to model more intricate relationships [25], [49].

## 4 Personalized health impact prediction

Conventional research on tobacco-induced health risks faces significant gaps in the personalized health impact prediction from tobacco use. This could be effectively addressed by harnessing the power of ML, which can analyze vast datasets to identify biomarkers and genetic profiles predisposing individuals to diseases like lung cancer and cardiovascular conditions, generate personalized predictions of adverse smoking outcomes, enhance information extraction from scientific literature, and forecast long-term health outcomes to guide public health interventions and policies [50]. By using ML techniques, researchers identified specific gene isoforms that are significantly different between current and former smokers [51]. These isoforms were identified through a process that included feature selection and ranking algorithms, leading to the development of classification models that can effectively differentiate between the two groups. The identified isoforms and pathways provide insights into the biological impact of smoking, which can help in developing targeted personalized interventions and treatments for smoking-related diseases. ML is also being used to identify specific pathways through which smoking causes damage, especially in the presence of certain comorbid conditions. Toon et al. applied ML to analyze proteomic data from cultured bronchial epithelial cells exposed to cigarette smoke extract, identifying ferroptosis as the most distinctive and significantly affected pathway in COPD patients compared to non-COPD individuals [52]. ML-based feature selection enabled the identification of this key pathway, highlighting the particular vulnerability of COPD epithelial cells to smoke and advancing our understanding of COPD pathogenesis. This could help start personalized treatment plans for COPD patients who smoke.

Non-communicable disease (NCDs) account for 70 % of global deaths, with tobacco as a key cause [53]. Smoking undermines the UN's Sustainable Development Goals, but cessation could reduce the NCD burden by one-third by 2030 [54]. Smoking Induced Non-Communicable Diseases (SiNCDs) such as stroke, heart disease, chronic respiratory diseases [55], cancers, etc., can have significantly reduced death rates through early diagnosis, effective treatment, and smoking cessation [56], [57]. ML demonstrates significant potential in predicting various aspects of the likelihood of SiNCDs, which we will discuss in the next section.

### 4.1 SiNCDs

ML is being utilized to predict the likelihood of developing SiNCDs accurately [58]. The study proposed an effective XGBoost framework integrated with a hybrid feature selection (HFS) technique for SiNCD prediction [58]. This XGBoost framework was applied to real-world NHANES datasets from South Korea and the United States [58]. Comparative analysis showed that the proposed model not only addressed the drawbacks of traditional studies in accurate prediction of SiNCDs in a personalized manner but also outperformed existing baseline models.

#### 4.1.1 Predicting lung cancer

Lung cancer, a major SiNCD, a leading cause of cancer deaths, is strongly linked to prolonged tobacco exposure, with risk increasing with more years of smoking [59]. Most studies focus on the genetic and biological mechanisms connecting smoking to lung cancer. However, many long-term smokers never develop lung cancer, indicating complex interactions and body responses to tobacco [60]. Additionally, lung cancer also occurs in never-smokers, often diagnosed at late stages. Two significant gaps in traditional studies are evident: the need to identify risk pathways of smoking carcinogenesis for early lung cancer diagnosis and personalized treatment in smokers, and the necessity to assess lung cancer risk based on smoking status, especially for never-smokers [61]. Researchers are harnessing ML to address these gaps by providing more sophisticated and accurate risk assessment models [62]. Chen and Lin investigate the impact of smoking factors on lung cancer risk and identifies specific risk pathways associated with smoking-induced lung cancer using ML [60]. They identified five optimal feature pathway sets, which, when combined with clinical information, like gender, age, smoking index, lymphatic lesions, etc, improved diagnostic accuracy to 90 % using a SVM model [60]. The study highlights the potential of the identified pathways as effective risk indicators for differentiating between lung cancer smokers and non-lung cancer smokers, demonstrating their utility in enhancing diagnostic accuracy in clinical settings [60]. Nemlander et al. performed a study which examines how symptoms reported via an adaptive e-questionnaire predict lung cancer across never smokers, along with former and current smokers [61]. Stochastic gradient boosting, stratified by smoking status, was employed to train and test predictive models [61]. Key predictors were age, sex, and education level. This study developed ML-based risk assessment models that could become valuable clinical tools for evaluating lung cancer risk especially in never smokers.

#### 4.1.2 Predicting smoking-related postmenopausal osteoporosis

Osteoporosis, a skeletal disorder marked by reduced bone strength and density, is particularly prevalent in post-menopausal women, with over 50 % developing post-menopausal osteoporosis (PMOP) due to hormonal changes [63]. However, the condition occurs twice as often in women who smoke compared to non-smokers, resulting in smoking-related postmenopausal osteoporosis (SRPO) [64]. High-throughput microarray technologies have revolutionized disease research by pinpointing genomic variations, complemented by weighted gene co-expression network analysis (WGCNA) to link gene modules with disease traits [65]. Applying these advancements to SRPO research holds significant promise, yet traditional methods struggle with the complexity of such large datasets. ML could bridge this gap, enhancing SRPO research by efficiently handling complex, high-dimensional data [66]. ML algorithms integrate genomic and transcriptomic data to pinpoint specific genetic variations and biomarkers [67]. Techniques like deep learning and ensemble methods improve predictive accuracy by uncovering intricate relationships among variables. Furthermore, ML has the potential to reveal new therapeutic targets by analyzing gene expression data to identify crucial pathways and networks relevant to SRPO [68]. Continuous adaptation and learning from new data enable ML models to provide ongoing insights, driving forward our understanding and treatment of SRPO. Using ML methods SVM-RFE and RF feature selection, Li et al. explored biomarkers and molecular pathways in SRPO, pinpointing six genes (HNRNPC, PFDN2, PSMC5, RPS16, TCEB2, UBE2V2) as genetic indicators [69]. Their findings not only identified potential biomarkers but also offered new insights into the molecular mechanisms driving SRPO. These findings offer specific genetic biomarkers (HNRNPC, PFDN2, PSMC5, RPS16, TCEB2, UBE2V2) for SRPO, which can advance traditional research by guiding further studies into disease mechanisms and facilitating targeted therapies. Clinically, these biomarkers could enhance early detection and personalized treatment strategies, improving outcomes for SRPO patients and potentially leading to the development of new diagnostic tools and therapies.

ML techniques have been effectively used to identify biomarkers and genetic profiles associated with smoking-related diseases. For instance, XGBoost has been employed to predict the likelihood of SiNCDs and identify specific gene isoforms linked to smoking. There is a need for more comprehensive studies that incorporate diverse populations to validate the predictive models further and assess their applicability in real-world settings. Future work could explore deep learning techniques to analyze complex genomic data, potentially leading to the discovery of novel biomarkers for personalized interventions. Additionally, integrating multi-omics data (genomics, proteomics, etc.) using ensemble methods could enhance the predictive accuracy of health outcomes.

## 5 Understanding passive smoke exposure

Secondhand and thirdhand smoke exposure can be classified as inactive or involuntary smoking, which pose significant public health risks, linked to respiratory infections, cardiovascular diseases, and cancer [70]. Secondhand smoke (SHS) is smoke that non-smokers involuntarily inhale from nearby smokers, whereas thirdhand smoke (THS) comprises residual contaminants that linger on surfaces and dust. Children are particularly vulnerable to exposure to both SHS and THS, posing significant health risks [71].

Traditional methods like observational studies and self-reported surveys, despite extensive research, suffer from accuracy issues, recall bias, high costs, and inefficiency with large datasets. ML offers promise by enhancing prediction of infant tobacco smoke exposure through advanced data analysis, improving understanding of less apparent sources like THS and less apparent routes like oral or dermal routes. Parks et al. highlighted ongoing challenges in reducing smoke exposure and the need for further exploration of SHS/THS impacts [71]. The study evaluated how well questionnaires could predict changes in urinary cotinine and 3HC levels. It found that the pervasiveness and persistence of the SHS and THS is so profound that modeling also could not predict more than half of the variation in urinary cotinine and 3HC levels in infants [71]. Despite low maternal smoking rates, high levels of cotinine (76 %) and 3HC (89 %) were detected in infants' urine, with questionnaire models explaining up to 41 % of variance [71]. Identified cut-points suggest significant SHS exposure in 23.5 % of infants [71]. Also, while THS is a recognized public health risk, traditional methods lack reliable biomarkers to distinguish it from SHS, limiting accurate assessments. A study by Merianos et al. aimed to improve differentiation between SHS and THS exposure in children using ML [72]. The model achieved prediction accuracies of 100 % for no/minimal exposure, 88 % for predominant THS exposure, and 71 % for mixed SHS and THS exposure [72]. Key predictors included the number of household smokers, serum cotinine, serum hydroxycotinine, and urinary 4-(methylnitrosamino)-1-(3-pyridyl)-1-butanol [72]. This demonstrates that ML can effectively distinguish between SHS and THS exposures, enhancing public health strategies for protecting children.

Smoking during pregnancy significantly impacts infant health, increasing risks of NCDs through DNA methylation, with studies identifying 185 altered CpG sites linked to maternal smoking, highlighting potential targets for understanding and mitigating these effects [73]. Rauschert et al. used ML to develop a DNA methylation score to identify fetal exposure to maternal smoking, using adolescent DNA data [74]. Tested in multiple cohorts, the score showed high sensitivity and specificity, outperforming existing non-ML based scores, and serves as a potential biomarker for assessing long-term health impacts of maternal smoking.

In addition to direct physiological effects, SHS has psychological impacts on non-smokers. Traditional studies struggle to identify key factors behind these psychological effects. Kim et al. used ML, specifically light gradient boosting machine (LGBM), to pinpoint stress perception, health status, and quality of life as key predictors of SHS-induced depression [75]. These findings highlight potential targets for depression-preventive interventions during public health crises.

ML has shown promise in distinguishing between secondhand and thirdhand smoke exposures, with studies achieving high prediction accuracies using various algorithms, such as LGBM. The current research does not extensively cover the long-term health impacts of THS and the psychological effects of SHS on non-smokers. Future research could leverage ML to handle and analyze large volumes of data from environmental sensors, air quality monitors, and wearable devices, providing accurate, continuous measurements of SHS and THS exposure. This real-time monitoring would enhance our understanding of exposure patterns and identifies high-risk environments and populations. ML also have the ability to improves predictive capabilities, identifying important predictors, forecasting potential exposure scenarios based on historical data, environmental conditions, and behaviors, enabling proactive protection measures. Furthermore, ML could personalize interventions by analyzing individual exposure data and health outcomes, tailoring strategies to specific needs. For instance, personalized recommendations can reduce SHS and THS exposure in homes and communities.

## 6 Predicting and improving smoking cessation outcomes

Smoking cessation programs aim to reduce tobacco-related diseases, but success rates vary due to factors like motivation, support systems, nicotine dependence, and psychological aspects. In the U.S., 70 % of smokers attempt to quit, but only 7 % remain abstinent for over a year [76]. Though health care provider and patient discussion about quitting tobacco smoking have significant positive effect on helping patient to quit smoke, due to poor success rates, many physicians deprioritize smoking cessation discussions, creating a negative feedback loop [76], [77]. Understanding smoking behavior is crucial for effective interventions. Data-driven, personalized approaches can improve outcomes by predicting individual success and optimizing cessation services and nicotine replacement therapies. ML enhances these efforts by analyzing large datasets to identify patterns and correlations and tracking real-time data from wearable devices for personalized feedback. Predictive analytics forecast success and offer timely interventions, while reinforcement learning continuously improves strategies. Integrating ML leads to more effective, personalized interventions, enhancing success rates and public health. In a recent study, Issabakhsh et al. utilized ML to identify key determinants of smoking cessation using data from the PATH survey, achieving a prediction accuracy of 72 % for cessation between waves 1 and 2, and 70 % for cessation between waves 2 and 3 [78]. ML techniques, including random forest, gradient boosting machines, and the SHapley Additive explanation method, were employed to select important variables and assess their impact. The analysis found that factors such as recent e-cigarette use, lower prior cigarette use, older age at smoking initiation, shorter smoking duration, poly tobacco use, and higher BMI were significant predictors of successful smoking cessation.

### 6.1 Identifying high risk population for tobacco uses

Smoking behavior has a significant genetic component, with heritability as high as 50 %. Predicting individuals' predisposition to smoking through genomic profiles could help prevent those at risk from starting. Using ML methods, primarily SVM and RF, Xu et al. built models to predict smoking behavior from single nucleotide polymorphisms (SNPs) data, achieving high accuracy with a model that combined logistic and LASSO regression for feature selection [79]. The studies by Lai et al. and Hu et al. complement the previous study by offering personalized, precision medicine for smoking cessation [80] and providing data-driven insights into key factors in smoking discussions between health providers and patients [77]. These approaches can potentially improve success rates for individuals attempting to quit smoking. ANN-based ML prediction model has been used to readily available patient data to provide reliable, individualized smoking cessation success rates, thereby enhancing shared decision-making between physicians and patients [80]. Using data from 4875 patients at a medical center in Northern Taiwan, the model predicted smoking cessation outcomes over six months [80]. Thus, knowing both genetic predisposition and the likelihood of cessation success can greatly enhance the efficacy of smoking cessation programs. Bayesian Additive Regression Trees (BART), a ML technique, to recognize health care resource usage, smoking intensity and duration and smoking-related conditions were key determinants promoting discussions on smoking and facilitating smoking cessation, whereas the “usual suspects”, age, gender, race and ethnicity were less important, and gender, in particular, had little effect on the likelihood of provider-patient discussions about smoking [77].

### 6.2 Predicting different aspects of smoking behavior

In a smoking cessation-related study, ML has been harnessed through a CNN-LSTM-based deep learning architecture named DeepPuff, which quantifies Respiratory Smoke Exposure Metrics (RSEM) by detecting smoke inhalations via breathing and hand gesture sensors from the Personal Automatic Cigarette Tracker v2 (PACT 2.0) [81]. The model achieved high precision in identifying smoke inhalations and provided reliable metrics for smoke exposure, comparable to those obtained through video annotation. The outcome demonstrates that the ML model, DeepPuff, is a reliable tool for measuring smoke exposure under free-living conditions, significantly contributing to the detailed assessment of smoking behavior and its health effects. In another study by Le et al., ML techniques, specifically RF paired with Recursive Feature Elimination and XGBoost, were used to identify predictors of smoking initiation among adults using the PATH study [82]. Notably, BMI and dental and oral health status emerged as robust predictors of smoking initiation, alongside other established risk factors. This research highlights the effectiveness of ML in uncovering new predictors and enhancing our understanding of tobacco use behaviors. Evans et al. utilized ML to identify and analyze distinct smoker profiles from a national survey, revealing four key groups: high-risk alcohol drinkers without children; single individuals in social housing with poor health and mental health; younger singles who have tried e-cigarettes and have poor mental health; and older couples with poor health [83]. These profiles differed notably from those of ex-smokers, who generally had better affluence, employment, and were older. This segmentation helps tailor targeted interventions and policies to specific smoker demographics, enhancing the effectiveness of smoking cessation efforts. Thakur et al. developed a ML-based framework using data from a wrist-wearable IMU sensor to accurately detect smoking activity among daily activities in real-time, achieving up to 98.7 % accuracy [84]. This ML approach enables just-in-time interventions for smoking cessation, aiding healthcare professionals in monitoring and supporting patients' quit efforts.

ML holds significant promise in advancing tobacco research, particularly in understanding the distinct epigenomic markers associated with different forms of smoking. A recent study on a Middle Eastern population, including never-smokers, cigarette-only smokers, and waterpipe-only smokers, used DNA methylome-wide profiling to reveal predominantly unique epigenetic markers for waterpipe smokers, distinct from those of cigarette smokers [85]. Remarkably, ML algorithms could accurately infer smoking forms with about 90 % accuracy based on DNA methylation patterns. These markers exhibited dose–response relationships with smoking extent and were validated through additional samples and independent technologies. By identifying markers enriched in regulatory regions and addiction-related pathways, the study highlights ML's potential to uncover specific epigenetic changes linked to addiction, aiding in the development of targeted interventions and prevention strategies for tobacco-related illnesses.

As mentioned in earlier section that vaping mostly associated with several risk factors, some research indicates that certain traits may be linked to adult smokers who successfully quit using e-cigarettes [86]. A study involving 889 adult smokers in Ontario used a gradient boosting machine model to identify key predictors of success in vaping-assisted smoking cessation [86]. The model, which achieved high performance with an ROC curve of 0.865 and a classification accuracy of 0.831, found that positive vaping experiences, fewer prior failed quit attempts, younger age, frequent vaping, and vaping shortly after waking were top predictors of perceived success [86]. This type of ML model could assist healthcare providers in recommending vaping for smoking cessation when it is likely to be beneficial. However, due to the risks of nicotine dependence and other health issues, vaping should be strictly limited and carefully considered, especially for adolescents.

### 6.3 ML powered apps

The low success rate in tobacco cessation is concerning. Nicotine cravings are the primary driver of smoking urges, but factors such as location, time, the presence of other smokers, and specific activities also play a significant role in triggering these urges. Studies show higher cravings in the mornings, evenings, at home, bars, and during media consumption. Effective cessation requires timely, targeted interventions. Smoking cessation apps use data from smartphones and smartwatches to track these factors, providing just-in-time adaptive interventions (JITAI) through SMS, calls, or notifications, offering a cost-effective alternative to traditional methods [87]. Here, ML can enhance smoking cessation efforts by using real-time, environmentally contextual data to tailor interventions through Ecological Momentary Assessment (EMA) [88], [89]. Hebert et al. recently unrevealed strong predictors of first lapse during quit attempts [90]. Through the elastic net algorithm, the study presented five strongest predictors (i.e., perceived odds of smoking today, motivation to avoid smoking, confidence in ability to avoid smoking, cigarette availability, and urge to smoke) of the first lapse during quit attempt [90]. Similarly, Koslovsky et al. showed that Bayesian structural time series as a vigorous method to evaluate the risk factors connected with various stages of the tobacco smoking cessation process [88]. The model was effective in identifying 20 predictors (like, availability of cigarette, craving to smoke, location, behavioural and environmental factors etc.), their associations and their influence during smoking to non-smoking transition state or vice a versa. Dumortier et al. used Bayes, decision tree learning, and discriminant analysis on data from 349 smokers to accurately classify high craving states during quit attempts, highlighting ML's potential to aid smoking cessation and forecast urges via mobile health apps [91]. Another related recent study by Abo-Tabik et al. developed a deep learning-powered app that learns a smoker's routine and predicts smoking events. This app adjusts for individual differences among smokers and uses motion and geolocation data collected from mobile devices to forecast smoking occurrences [92]. However, it can be conclusively said that, combining passive automated data collection with ML models can enhance smoking cessation apps by providing just-in-time adaptive interventions during high-risk moments, thereby reducing lapses and preventing relapse, while researchers should prioritize high-quality sensor data over unreliable self-reported data.

In a recent study, Huang et al. harnessed ML to identify predictors of success in a depression-specific smoking cessation intervention using the Goal2Quit app [93]. By employing the LASSO to analyze baseline variables, the study determined factors such as time spent using the app and educational attainment [93]. Results showed that significant app usage and having an educational degree beyond high school were predictive of successful smoking reduction and depression improvement. This approach allowed for the personalization of digital cessation interventions, highlighting the importance of tailoring treatments based on user engagement and educational level.

A recent interesting study how ML can be utilized to play a critical role in this smoking cessation-related study by using supervised ML (SML) algorithms to analyze user data from a smoking cessation app [94]. The study aimed to identify app features that promote successful smoking cessation. By recording participants' app activity, demographic data, and tobacco use behaviors, the SML model was trained to predict the likelihood of cessation based on the use of different app features. The SML model demonstrated reasonable accuracy in predicting cessation, highlighting that patterns of app feature use could account for variance in smoking cessation beyond known predictors. This approach shows the potential of ML to optimize and enhance the effectiveness of smoking cessation apps.

Vera et al. used ML to identify key predictors of engagement with the Stop-Tabac smoking cessation app [95]. Algorithms helped reveal that the top predictors included the user's intention to stop smoking, dependence level, perceived helpfulness of the app, quitting success after one month, app usage after one month, group assignment (experimental vs. control), age, and years of smoking [95]. These insights can help tailor the app to user needs, improve enrolment, and enhance content. Etter et al. used ML algorithms to analyze data from 5293 daily smokers using the Stop-Tabac app, identifying key predictors of smoking cessation, reduction, and relapse at six months [96]. ML helped identify that tobacco dependence, motivation to quit, frequency of app use, perceived app usefulness, and nicotine medication use were significant predictors of smoking outcomes. This research provides valuable insights for improving smoking cessation apps and guiding future studies.

ML has been utilized to enhance smoking cessation outcomes by analyzing large datasets to identify key predictors of success. For example, Issabakhsh et al. achieved significant prediction accuracy using Random Forest and GBM. There is a lack of integration of behavioral and psychological factors into ML models, which could provide a more holistic understanding of smoking cessation. Another study by Perski et al. highlighting the need for combining unprompted and prompted data for effective intervention development [97]. This study used app-based data to develop ML models for predicting smoking lapses. Group-level models performed well (AUC = 0.969) but showed variability with new users [97]. Individual and hybrid models improved accuracy but were limited by data availability. Future studies could implement hybrid models combining ML with psychological theories to better predict cessation success. Additionally, exploring reinforcement learning for adaptive intervention strategies could lead to more effective cessation programs tailored to individual needs.

## 7 Effective intervention in the evolving smoking tobacco product landscape

Nicotine poses significant health risks, not only through traditional combustible products like cigarettes, cigars, and pipes, but also through evolving new age tobacco products like electronic nicotine delivery systems (ENDS), commonly known as vapes [98]. Experts considering the phenomenon as vaping epidemic [99]. Marketed under various names like e-cigarettes, vape pens, and pods by brands such as JUUL and Puff Bar, these devices heat nicotine-containing e-liquids to produce an aerosol for inhalation, mimicking smoking [100]. Despite their initial intent for smoking cessation, they've become popular among youth, with over 2.1 million U.S. teens currently using them [101]. This trend is alarming due to nicotine's adverse effects on attention, memory, and learning, potentially leading to addiction and subsequent tobacco use. Moreover, vapes contain harmful additive substances, toxic metals and chemicals similar to those in combustible cigarettes, contributing to irreversible lung damage [102]. The 2019 outbreak of vaping-associated pulmonary injury (EVALI) further underscores these risks, with numerous cases reported globally [103]. New-age tobacco products can be effectively managed using several ML approaches. ML can identify predictors for adolescent ENDS addiction, enhancing the precision of prevention and intervention strategies. Text-based sentiment analysis from social media can gauge public perceptions and concerns about these products, guiding targeted public health campaigns. ML can analyse online advertisements to detect and regulate misleading or youth-targeted promotions, thereby strengthening advertising regulations. Additionally, ML can detect toxic substances in ENDS, aiding regulatory efforts to ensure product safety and mitigate health risks associated with their use. These approaches are essential because traditional methods struggle with the vast volume of data involved, limiting their ability to derive timely and comprehensive insights. ML's capability to process and analyse big data enables efficient evaluation of predictors, sentiment analysis, advertisement content, and toxicity detection, significantly enhancing tobacco control efforts.

### 7.1 Identifying toxic substances in ENDS

Recent studies also highlight ML’s promise in identifying the toxic effects of vaping when combined with traditional approaches. Vaping heats e-liquids to high temperatures, potentially creating harmful decomposition products. Kishimoto et al. uses a graph-convolutional neural network model predicted pyrolysis reactivity for 180 e-liquid flavors, generating 7307 products, which were refined using mass spectrometry data to identify 1169 molecular weight matches, revealing numerous toxic and hazardous compounds, thus aiding in understanding vaping's long-term health risks [104]. ML, particularly the CatBoost algorithm, enhances detection of nicotine-containing e-liquids via surface-enhanced Raman scattering (SERS) [102]. This approach improves sensitivity and accuracy, enabling rapid on-site screening with portable devices and complementing central lab analyses. It holds potential for identifying prohibited additives, advancing tobacco control efforts.

### 7.2 Factors influencing youth smoking initiation

Intervention strategies aimed at preventing teenage ENDS use should target specific predictors unique to these products, such as digital media engagement and technology interest. Han et al. employed supervised ML, particularly penalized logistic regression, to identify these predictors from the Population Assessment of Tobacco and Health Study (PATH) [19]. Their findings highlight that frequent social media use significantly predicts ENDS use, distinct from other substance use behaviors, suggesting the influence of ENDS marketing on social platforms. Moreover, social media use predicts future cigarette smoking and experimentation with substances like alcohol and marijuana, underscoring broader risks associated with ENDS use. Recent studies further reinforce the impact of social media and identify additional predictors among tobacco-naive young adults, including susceptibility to ENDS, physical exercise, marijuana use, and susceptibility to cigarette use [105]. Shi et al.'s use of random forest algorithms supports this substance use predictors and reveals new insights such as the influence of school absences and significant ethnic interactions in predicting ever-vaping [106]. Vazquez et al. utilized the elastic net algorithm to validate individual and socioecological classifiers previously identified, such as substance use behaviors, perceptions of e-cigarette availability and risk, school-related factors like suspensions, and social influences like friends' behaviors [107].

Singh et al. used ML to identify predictors of vaping dependence over three months among daily and non-daily adolescent vapers. Key predictors for daily vapers included purchase location, pod usage duration, and nicotine vaping frequency; for non-daily vapers, predictors included race, sexual orientation, and heart disease treatment [108]. Fu et al. employed random forest to predict frequent vaping among adolescents, highlighting predictors such as higher past-month nicotine concentration in vape, frequent daily vaping sessions, and greater nicotine dependence [109]. These studies emphasize the importance of socioecological factors such as age, perceived discrimination, and race/ethnicity in shaping adolescent attitudes towards vaping. Le also highlighted the influence of peer behaviors, household tobacco use, curiosity about ENDS, and perceptions of product safety [110].

These studies clearly indicate that social media usage and socioecological factors such as school-related issues, peer influence, ethnicity, and family attitudes toward tobacco play critical roles as predictors of adolescent ENDS use. These insights underscore ML's role in informing precise prevention strategies amid increasing ENDS use among youths, emphasizing the impact of family and social environments on adolescent tobacco behaviors. They reveal previously unknown insights and demonstrate ML's potential to enhance ENDS prevention efforts by swiftly analyzing complex socioecological variables, aiding in early intervention for at-risk youth in the growing e-cigarette epidemic. These ML insights can significantly contribute to targeted and effective tobacco control policies.

### 7.3 Predicting tobacco trends from social media data

#### 7.3.1 Sentiment analysis from social media

Text-based sentiment analysis from social media helps prevent the vaping epidemic by monitoring public opinions and detecting trends in e-cigarette use. But traditional studies on public attitudes toward vaping are hindered by manual data identification. ML algorithms are able to handle this big data and can extract meaningful insights by analyze posts and comments. It can identify shifts in sentiment and misinformation, allowing authorities to respond promptly and tailor interventions. This real-time data shapes effective tobacco control policies and discourages vaping, especially among youth.

Hassan et al. used text mining of tweets during the EVALI outbreak to analyze public sentiment, demonstrating the effectiveness of ML in extracting insights relevant to tobacco control policy [111]. These methods can inform regulatory policies for ENDS. A study by Ren et al. used a stacking ensemble model that accurately identified vaping-related tweets with an F1-score of 0.97 [112]. They showed that how ML automation enhances the efficiency and insightfulness of analyzing social media data for public opinion and health surveillance in respect to new age tobacco product.

ML also have the ability to popularize the anti-vape opinion on social platform as shown by a very recent study [113]. Xie et al. demonstrates how ML, particularly deep-learning models and statistical techniques, enhances tobacco research by analyzing anti-vaping Instagram image posts to identify features associated with high user engagement [113]. By employing advanced ML algorithms, the study identified key image features and textual content that significantly correlate with increased likes and comments on anti-vaping posts. These findings highlight ML's capability to extract nuanced information from large datasets, offering insights into effective communication strategies for public health messaging on e-cigarette risks. By addressing the prevalence of pro-vaping content on social media, this research underscores the importance of leveraging ML to shape targeted interventions aimed at curbing youth vaping and promoting public health awareness.

#### 7.3.2 Surveillance on online advertisement across popular sites

Surveillance of online advertisements helps prevent the vaping epidemic by identifying and removing youth-targeted marketing, ensuring compliance with advertising laws, and reducing exposure to pro-vaping messages. Increased scrutiny of youth vaping highlights social media's role, with unsupervised ML analysis of 70,725 Instagram posts revealing 3331 ENDS sales, primarily by individuals and retailers, often without age verification, underscoring the need for stricter enforcement [114]. ML, particularly DL classifiers like LSTM-CNN, shows superior performance in identifying and characterizing vaping-related tweets without requiring extensive annotations [115]. This capability supports the development of an effective X-based vaping surveillance system, overcoming the limitations of traditional classifiers. E-cigarette promotion on social media, especially JUUL pod vaporizers, surged alongside youth e-cigarette use. Kostygina et al. analyzed JUUL-related messages on Instagram using ML algorithms, finding that most commercial posts used recruitment and addiction appeals, rather than cessation-related messaging [116]. The study by Kong et al. effectively utilizes ML to analyze e-cigarette content on YouTube, identifying prevalent themes like product reviews and instructional videos, prominent e-cigarette products, and various marketing strategies such as discounts and sales promotions [117]. Murthy et al. illustrates how ML, specifically computer vision techniques like YOLOv7, contributes to tobacco research by effectively detecting e-cigarette-related content in visual media on TikTok [118]. By automating the detection of vaping devices, hands, and vapor clouds from a dataset of TikTok images, the model achieved high accuracy and recall rates, demonstrating its robustness in identifying e-cigarette imagery. Another very recent study showcases the efficacy of deep learning-based object detection in monitoring e-cigarette product presence across Instagram and TikTok. Using a DyHead model with a Swin-Large backbone, researchers accurately identified various e-cigarette-related objects in images and videos, revealing increasing trends in promotional content over time [119]. This automated approach offers scalable surveillance capabilities, crucial for informing tobacco regulatory science and aiding social media platforms in promptly moderating tobacco-related imagery to mitigate adolescent exposure online.

A recent study by Lakatos et al. even harnesses ML to uncover hidden threats regarding covert advertisements of tobacco [120]. This study shows promise in detecting covert tobacco advertisements by enabling unbiased and reproducible analysis of tobacco-related media even increasing scope to strengthening the regulatory measures. An integrated model combining text and image processing, generative techniques, and human reinforcement achieves detection accuracies of 74 % for images and 98 % for text [120]. This approach leverages pre-trained multimodal deep learning models to identify smoking content across media formats, even with limited training data, while allowing expert intervention for enhanced accuracy.

These findings highlight how ML-driven computer vision systems can improve surveillance and regulatory efforts by quickly identifying and analyzing e-cigarette content on popular social media platforms. ML models accurately categorize video content and detect sales-related themes, which is vital for monitoring trends and understanding youth exposure to vaping imagery [120]. These information helps in developing targeted public health interventions to reduce the impact of e-cigarette use among young people. It also underscores the urgency of implementing stricter regulations to counter persuasive e-cigarette marketing aimed at youth online.

Based on current trends and research, it is reasonable to predict that ML will significantly address ENDS-related public health issues. By analyzing data from social media, sales platforms, and health records, ML can monitor trends, detect non-compliant products, assess health risks like EVALI, and provide personalized smoking cessation interventions. This will likely enhance regulatory oversight and inform effective decisions on product formulations, marketing claims, and health impacts, thereby improving public health responses to ENDS.

## 8 Economic impact of tobacco smoking

Nargis et al. estimated state-level economic losses attributable to cigarette smoking in the USA using a dynamic macroeconomic model of personal income per capita, analyzing data from 2011 to 2020 with a mixed-effects, generalized linear, dynamic panel data model [121]. The study found substantial income losses, with a national annual combined loss of $436.7 billion and a cumulative loss of $864.5 billion in 2020 due to smoking [121]. While this study did not use ML, ML could enhance such economic models by analyzing large datasets more effectively, uncovering hidden patterns, refining estimates, and enabling real-time adjustments as new data becomes available. Smoking leads to significant economic losses in the USA. Equitable tobacco control measures can greatly enhance macroeconomic performance both short and long term by lowering health costs and preventing productivity declines.

## 9 Conclusion

ML has revolutionized tobacco research by providing innovative solutions to the complexities of tobacco use and its associated health impacts. With its ability to analyze extensive datasets and apply advanced algorithms, ML uncovers patterns, predicts outcomes, and personalizes interventions. This capability enhances our understanding of tobacco-related health risks and improves cessation strategies. Significant insights driven by ML include the identification of genetic predispositions to smoking-related diseases, predictions of smoking behaviors, and assessments of secondhand and thirdhand smoke exposure. These findings not only deepen our comprehension of the biological mechanisms underlying tobacco addiction but also aid in developing targeted and personalized interventions that can lead to improved health outcomes. Despite these advancements, ML in tobacco research faces challenges. There is a lack of personalized health impact predictions for SiNCDs beyond lung cancer and SRPO, despite the existence of numerous other SiNCDs that should also be considered for prediction using ML models in the smoking population. Also on the technical side, the effectiveness of ML models depends on high-quality data; inaccurate or biased datasets can lead to misleading results and ineffective interventions. Additionally, many ML models, especially deep learning algorithms, are difficult to interpret, which complicates understanding their decision-making processes. Ethical issues regarding privacy, consent, and data misuse also arise. To address these challenges, researchers should focus on improving data quality, fostering institutional collaborations for better data sharing, enhancing model transparency through interpretable techniques, and establishing clear ethical guidelines for data use and privacy. Despite these drawbacks, the future of ML in tobacco research appears promising. The integration of wearable devices and environmental sensors can provide continuous data on tobacco exposure, enabling real-time monitoring and personalized interventions. ML algorithms will increasingly support the development of tailored cessation programs that adapt to individual behaviors and health profiles. Additionally, ML can assist in monitoring and regulating emerging tobacco products, such as e-cigarettes, by analyzing social media trends and sales data to identify potential public health risks. Future research will benefit from cross-disciplinary collaboration among data scientists, public health experts, and clinicians, fostering innovation and developing comprehensive strategies to combat tobacco use. In conclusion, the integration of ML into tobacco research signifies a paradigm shift, offering advanced tools to understand and address tobacco use. Embracing ML while addressing its challenges can enhance public health outcomes and advance the global fight against tobacco use in a responsible and ethical manner.

Future investigations in tobacco research should focus on adopting cutting-edge ML techniques to uncover untapped opportunities and enhance current methodologies. For predictive tasks, transformer-based models like Vision Transformers (ViTs) and Temporal Fusion Transformers (TFTs) can process diverse datasets to predict disease risks and understand patterns of health decline over time [122], [123]. Behavioral analyses could utilize unsupervised methods, such as Variational Autoencoders (VAEs), to segment smoker profiles, while reinforcement learning strategies like deep Q-networks (DQN) and proximal policy optimization (PPO) could help predict and influence behavioral changes in response to interventions [124], [125]. Moreover, advanced computer vision algorithms, including Vision Transformers, can significantly improve the detection of adulterants and counterfeit tobacco products. Genomic research can benefit from Graph Neural Networks (GNNs) to examine intricate gene-environment interactions and facilitate the development of nicotine addiction treatments. Public health studies can employ sophisticated natural language processing models, such as GPT-4, for monitoring tobacco-related discussions on digital platforms and utilize causal ML methods like Structural Equation Models (SEMs) to assess the outcomes of policy interventions [126], [127]. By incorporating Explainable AI and fairness-focused frameworks, these ML applications can promote ethical, transparent, and impactful advancements in tobacco research.

## Funding

No funding was received for the study.

## CRediT authorship contribution statement

**Nabanita Ghosh:** Writing – review & editing, Writing – original draft, Visualization, Validation, Software, Investigation, Conceptualization. **Krishnendu Sinha:** Writing – review & editing, Writing – original draft, Visualization, Validation, Supervision, Software, Project administration, Investigation, Conceptualization. **Parames Sil:** Writing – review & editing, Writing – original draft, Visualization, Validation, Supervision, Investigation, Conceptualization.

## Declaration of Generative AI and AI-assisted technologies in the writing process

During the preparation of this work the author(s) used freely available LLMs in order to improve language and readability, with caution. After using this tool/service, the author(s) reviewed and edited the content as needed and take(s) full responsibility for the content of the publication.

## Declaration of Competing Interest

The authors declare that they have no known competing financial interests or personal relationships that could have appeared to influence the work reported in this paper.

## Data Availability

Data will be made available on request.
